# Analysis of Inflammatory Mediator Profiles in Sepsis Patients Reveals That Extracellular Histones Are Strongly Elevated in Nonsurvivors

**DOI:** 10.1155/2021/8395048

**Published:** 2021-03-17

**Authors:** Tanja Eichhorn, Ingrid Linsberger, Lucia Lauková, Carla Tripisciano, Birgit Fendl, René Weiss, Franz König, Gerhard Valicek, Georg Miestinger, Christoph Hörmann, Viktoria Weber

**Affiliations:** ^1^Christian Doppler Laboratory for Innovative Therapy Approaches in Sepsis, Danube University Krems, Krems, Austria; ^2^Department for Biomedical Research, Center for Biomedical Technology, Danube University Krems, Krems, Austria; ^3^Institute for Medical Statistics, Center for Medical Statistics, Informatics and Intelligent Systems, Medical University Vienna, Vienna, Austria; ^4^University Hospital St. Pölten, Department for Anaesthesiology and Intensive Care, St. Pölten, Austria

## Abstract

The timely recognition of sepsis and the prediction of its clinical course are challenging due to the complex molecular mechanisms leading to organ failure and to the heterogeneity of sepsis patients. Treatment strategies relying on a “one-fits-all” approach have failed to reduce mortality, suggesting that therapeutic targets differ between patient subgroups and highlighting the need for accurate analysis of the molecular cascades to assess the highly variable host response. Here, we characterized a panel of 44 inflammatory mediators, including cytokines, chemokines, damage-associated molecular patterns, and coagulation-related factors, as well as markers of endothelial activation in 30 patients suffering from renal failure in the course of sepsis. All patients received continuous veno-venous hemodialysis with either high cut-off filters or with standard filters, and mediators were quantified for all patients at the initiation of dialysis and after 24 h and 48 h. Mediator concentrations in individual patients ranged widely, demonstrating the heterogeneity of sepsis patients. None of the mediators correlated with SAPS III or TISS scores. The overall in-hospital mortality of the study population was 56.7% (57.1% *vs.* 56.3% for high cut-off *vs.* standard filter). The two filter groups differed regarding most of the mediator levels at baseline, prohibiting conclusions regarding the effect of standard filters *versus* high cut-off filters on mediator depletion. The elevation and correlation of damage-associated molecular patterns and markers of endothelial activation gave evidence of severe tissue damage. In particular, extracellular histones were strongly increased and were almost 30-fold higher in nonsurvivors as compared to survivors, indicating their diagnostic and prognostic potential.

## 1. Introduction

The definition of sepsis as life-threatening organ dysfunction caused by a dysregulated host response to infection emphasizes the significance of the nonhomeostatic host response and highlights the need for timely recognition and treatment of sepsis [1, 2].

The clinical course of sepsis is highly heterogeneous and is influenced by both pathogen-related factors (type and load of pathogen, virulence, and site of infection) and host-related factors (age, gender, genetic background, comorbidities, and lifestyle) [3–5]. Therapeutic approaches to target individual mediators, such as lipopolysaccharide (LPS) or proinflammatory cytokines, have failed to demonstrate convincing benefit in clinical trials so far [6–9]. Post hoc analysis indicated benefits for certain patient subgroups in a number of studies, but these effects were diluted across the study population due to its pronounced heterogeneity [10, 11].

Sepsis is initiated by the recognition of pathogens via pathogen-associated molecular patterns (PAMPs) on innate immune cells, triggering the release of cytokines and chemokines [12, 13]. Simultaneously, injured host cells secrete damage-associated molecular patterns (DAMPs), including histones, high-mobility group box-1 protein (HMGB-1), and extracellular matrix components, such as heparan sulphate, amplifying the inflammatory response [14]. Activated neutrophils release neutrophil extracellular traps (NETs), chromatin-based structures associated with antimicrobial peptides, histones, myeloperoxidase, and elastase. Excessive NET formation promotes tissue damage and activation of coagulation, as well as endothelial activation and loss of barrier function [15–17]. Furthermore, proinflammatory mediators may induce leukocyte apoptosis, resulting in immune suppression and inability to cope with the primary or with secondary infections [18, 19].

To target this imbalance and to support the restoration of immune homeostasis, approaches for extracorporeal immunomodulation including continuous veno-venous hemodialysis (CVVHD) with high cut-off hemofilters have been introduced [20]. In a previous study in 30 patients suffering from renal failure in the course of sepsis, we compared the depletion of IL-6, IL-8, IL-10, and TNF-*α* with high cut-off versus standard high-flux hemofilters and observed enhanced clearance of IL-6 and IL-8 by high cut-off *vs.* standard filters both *in vitro* and *in vivo.* This enhanced clearance however did not result in persistently reduced cytokine levels, presumably due to a dynamic release of cytokines over the course of treatment [21]. We further found that plasma samples from individual patients differed considerably with regard to their potential to induce endothelial activation in a cell culture model [22].

Here, we report on inflammatory mediator profiles in the study population, comprising a panel of 44 inflammatory cytokines, chemokines, growth factors, DAMPs, and endothelial activation markers, as well as coagulation-related parameters, which were analyzed both at the initiation of dialysis and over the course of treatment.

## 2. Materials and Methods

### 2.1. Study Design

Plasma samples from sepsis patients were obtained within a single-center, randomized, controlled clinical study with the primary aim of comparing the depletion of IL-6, IL-8, IL-10, and TNF-*α* during CVVHD using the high cut-off filter Ultraflux EMiC2 and the standard filter Ultraflux AV1000S, both from Fresenius Medical Care (Bad Homburg, Germany). Both EMiC2 and AV1000S are polysulfone-based filters with a surface area of 1.8 m^2^ and an approximate molecular weight cut-off of 40 and 30 kDa, respectively. The study was approved by the Ethics Committee of the University Clinic St. Pölten, Austria (GS4-EK-3/082-2012), and was performed in accordance with the Declaration of Helsinki. Based on *in vitro* data on the clearance of IL-6 with the high cut-off filter *vs.* the standard filter (4.7 ± 0.2*vs.*0.8 ± 0.4 ml/min) and on literature data showing average IL-6 levels of 360 ± 116 pg/ml in sepsis patients, a sample size of 15 patients per group was calculated to achieve a power of 80% (*α* = 0.05), assuming a standardized difference of Cohen's *d* = 0.90 between the two treatment groups. The results regarding the primary study aim have been published elsewhere [21]. Here, we characterized the plasma concentrations of 44 inflammatory mediators in the study population over the course of treatment, as detailed below.

### 2.2. Inclusion Criteria

Patients suffering from acute renal failure in the course of sepsis were included in this study and recruited from November 2013 to October 2015. Acute renal failure was diagnosed according to RIFLE criteria [23]. The diagnosis of sepsis was established on the basis of a proven or suspected infection and the presence of at least two of the following criteria: (i) temperature > 38°C or < 36°C; (ii) heart rate > 90 beats per min; (iii) respiratory rate > 20 breaths per min or partial pressure of carbon dioxide in arterial blood (PaCO_2_) < 32 mmHg; and (iv) white blood cell count > 12 x 10^9^/l, <4 × 10^9^/l, or > 10% band forms [24]. Patients younger than 18 years, pregnant patients, and patients with a contraindication for continuous hemodialysis were excluded. Thirty patients were included and randomized either to the high cut-off (EMiC2) group (*n* = 14) or to the standard (AV1000S) filter group (*n* = 16) [21].

### 2.3. Treatment and Sample Collection

In both groups, CVVHD was performed for 48 h at a blood flow of 200 ml/min, and citrate was used as an anticoagulant (Ci-Ca protocol, Fresenius Medical Care). At admission, SAPS III (Simplified Acute Physiology Score) and TISS (Therapeutic Intervention Scoring System) scores, leukocyte counts, comorbidities, and the sites of infection were recorded. Blood samples were drawn at the onset of the study (0 h) and after 24 h and 48 h into vacuette tubes containing citrate as the anticoagulant (Greiner Bio-One, Kremsmünster, Austria). All samples were immediately centrifuged (2,000 g, 15 min, 4°C), and the resulting plasma samples were stored at -80°C until further analysis.

### 2.4. Quantification of Inflammatory Cytokines, Chemokines, and Growth Factors

The Bio-Plex Pro™ human cytokine 27-plex bead array (Bio-Rad, Vienna, Austria) was used to quantify interleukin- (IL-) 1*β*, IL-1 receptor antagonist (IL-1ra), IL-2, IL-4, IL-5, IL-6, IL-7, IL-8, IL-9, IL-10, IL-12p70, IL-13, IL-15, IL-17A, interferon-gamma (IFN-*γ*), tumour necrosis factor-alpha (TNF-*α*), monocyte chemotactic protein-1 (MCP-1), macrophage inflammatory protein-1 alpha and beta (MIP-1*α*, MIP-1*β*), regulated on activation, normal T-cell expressed and secreted (RANTES), eosinophil chemotactic protein (eotaxin), interferon-inducible protein 10 (IP-10), granulocyte colony-stimulating factor (G-CSF), granulocyte-macrophage colony-stimulating factor (GM-CSF), basic fibroblast growth factor (bFGF), platelet-derived growth factor (PDGF), and vascular endothelial growth factor (VEGF). Plasma samples were diluted to 1 : 4 with sample diluent and analyzed according to the instructions of the manufacturer. Plasma concentrations of C-reactive protein (CRP), soluble CD14 (sCD14), growth arrest-specific gene 6 (Gas6), and soluble suppression of tumorigenicity 2 (sST2) were quantified by the enzyme-linked immunosorbent assay (ELISA; R&D Systems Minneapolis, MN). LPS-binding protein (LBP) was measured by ELISA (Biometec, Greifswald, Germany).

The hybcell antibody microarray (CubeDX, St. Valentin, Austria) was used to quantify procalcitonin (PCT) and cystatin C. The Bromocresol Green Albumin Assay (Sigma-Aldrich, St. Louis, MO) was used to quantify albumin.

### 2.5. Quantification of Endothelial Activation Markers

Plasma concentrations of the endothelial-associated proteoglycan endothelial cell-specific molecule-1 (ESM-1, endocan) were determined by ELISA (Lunginnov, Lille, France). Angiopoietin-1 (Ang-1) and Ang-2 were quantified by ELISA (R&D Systems).

### 2.6. Quantification of DAMPs

Extracellular DNA (ecDNA) was isolated from 100 *μ*l of plasma from septic patients using the QIAamp DNA Mini Kit (Qiagen, Hilden, Germany) according to the recommended protocol, and total ecDNA was quantified with a Qubit fluorometer and dsDNA HS Assay Kit (Thermo Fisher Scientific, Waltham, MA). Extracellular histones were determined as components of nucleosomes using the cell death detection ELISA (Roche, Mannheim, Germany), wherein a monoclonal anti-histone antibody is used as a catching antibody in combination with a monoclonal anti-DNA antibody conjugate for detection. A mixture of histones H1, H2A, H2B, H3, and H4 from the calf thymus (Roche) was used as a standard. High-mobility group box-1 protein (HMGB-1) was quantified by ELISA (IBL International, Hamburg, Germany).

### 2.7. Quantification of Coagulation-Related Parameters

Plasma concentrations of tissue factor (TF) were measured by ELISA (R&D Systems). Tissue factor activity was analyzed with the Actichrome TF assay (Sekisui Diagnostics, Stamford, CT).

### 2.8. Statistical Analysis

Descriptive results of continuous variables are expressed as median and 25^th^ to 75^th^ quartiles, except for clinical characteristics at baseline which are indicated as mean ± standard deviation (range). For calculation, values of mediators that remained below the detection limit of a given test were set equal to the lower detection limit. Differences in inflammatory mediator concentrations among survivors and nonsurvivors at baseline were analyzed by the nonparametric Mann-Whitney test. The same test was used to assess statistical differences in inflammatory mediator concentrations at baseline and after 48 h between the filter groups. Repeated measures two-way ANOVA followed by Bonferroni's multiple comparison test was performed to assess the filter (EMiC2 *vs.* AV1000S) and time effect (0 *vs.* 24 *vs.* 48 h). This analysis could not be performed for ecDNA, histone, sST2, TF, and TF activity due to the lack of data at 24 h and 48 h for these parameters. For correlation analysis, all parameters were log_10_-transformed to obtain a proportionally constant variation. Correlations among various biomarkers were tested for significance using the nonparametric Spearman correlation test. For all statistical tests, a value of *p* < 0.05 was considered to be statistically significant.

## 3. Results

### 3.1. Patient Characteristics

Thirty patients suffering from sepsis in the course of acute renal failure were included in the study. The demographic and clinical characteristics of the study population at baseline are summarized in Table 1. The predominant comorbidities of the study population were cardiovascular disease (60%), as well as diabetes and neurological disorders (30% each). The abdomen represented the primary site of infection (33%). The overall in-hospital mortality of the study population was 56.7% (57.1% *vs.* 56.3% for high cut-off *vs.* the standard filter, respectively). Age, gender, physiological scores (SAPS III, TISS), and leukocyte counts at admission did not significantly differ between survivors and nonsurvivors (Supplementary Table 1).

### 3.2. Inflammatory Mediator Concentrations

The baseline median and interquartile range of the 44 inflammatory parameters analyzed in this study are summarized in Table 2. Plasma levels of IL-33 remained undetectable for the majority of patients and were therefore excluded from statistical analysis.

The baseline median and interquartile range of the inflammatory mediators for survivors and nonsurvivors are summarized in Supplementary Table 2. Plasma samples from nonsurvivors showed 28-fold higher median baseline levels of histones, 7.6-fold higher median levels of IL-2, and 3.1-fold higher median levels of IL-10 as compared to those from survivors. MCP-1, IL-15, and GM-CSF were more than 2-fold elevated in nonsurvivors. Except for histones (*p* = 0.025), however, none of these differences reached statistical significance.

### 3.3. Correlation of Inflammatory Cytokines, Chemokines, and Growth Factors

**Figure 1:**
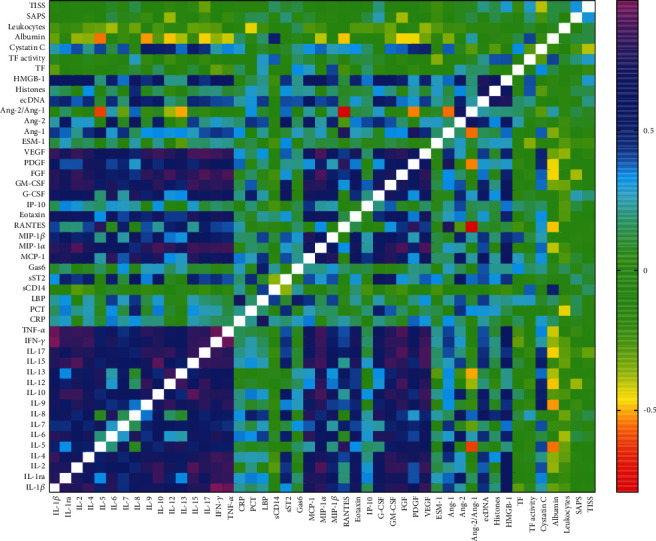
Correlation plot of all parameters determined in this study at baseline (0 h). The plot is based on the Spearman correlation between each pair of biomolecule. On the right, the Spearman correlation coefficient is indicated by the color gradient.

A plot of all parameters obtained by Spearman correlation analysis at baseline is shown in Figure 1. The strong correlation of IL-1*β*, IL-1ra, IL-4, IL-6, IL-8, IL-10, IL-15, IFN-*γ*, TNF-*α*, MCP-1, G-CSF, and GM-CSF confirmed the presence of a systemic, proinflammatory cytokine and chemokine response in the patient cohort, as detailed in Table 3. In particular, IL-1*β* correlated with both IFN-*γ* (*r* = 0.963, *p* < 0.0001) and TNF-*α* (*r* = 0.936, *p* < 0.0001), which also correlated with each other (*r* = 0.934, *p* < 0.0001). IL-15 and IL-10 were also strongly correlated (*r* = 0.912, *p* < 0.0001). We failed to detect a correlation between any of the biomarkers with disease severity, assessed by the SAPS III and TISS scores, except that SAPS III showed a weak negative association with IL-12 (*r* = −0.374, *p* = 0.042) and FGF (*r* = −0.035, *p* = 0.086).

### 3.4. Damage-Associated Molecular Patterns and Coagulation-Related Parameters

**Figure 2:**
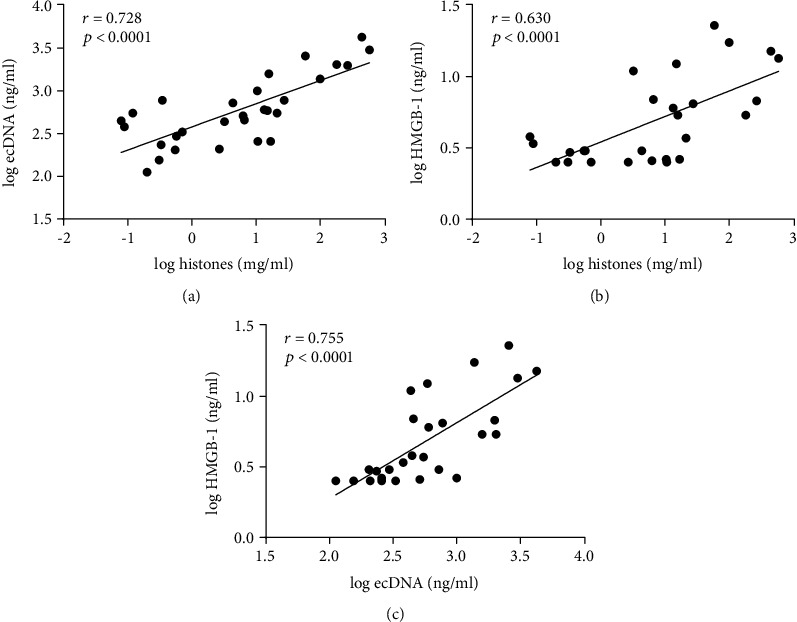
Correlation analysis of damage-associated molecular patterns in septic plasma samples. (a) Histones *vs.* extracellular DNA (ecDNA), (b) histones *vs.* high-mobility group box-1 protein (HMGB-1), (c) ecDNA *vs.* HMGB-1. The Spearman correlation test was used to calculate linear relationship between variables at baseline (0 h). Histones (*n* = 29), ecDNA (*n* = 29), and HMGB-1 (*n* = 27).

**Figure 3:**
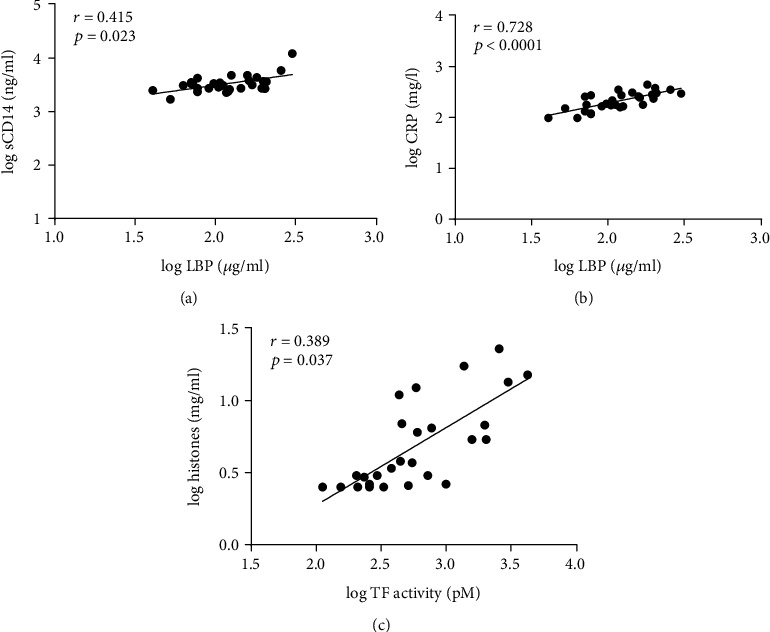
Correlation analysis of inflammatory mediators in septic plasma samples. (a) LBP *vs.* soluble CD14 (sCD14), (b) lipopolysaccharide-binding protein (LBP) *vs.* C-reactive protein (CRP), and (c) tissue factor (TF) activity *vs.* histones. The Spearman correlation test was used to measure the degree of association between variables at baseline (0 h; *n* = 30 for LBP, sCD14, CRP, and TF activity; *n* = 29 for histones).

To investigate the presence of damage-associated molecular patterns released from cells following tissue injury, we quantified extracellular DNA, histones, and HMGB-1, which significantly correlated with each other (Figure 2). In particular, the elevated levels of both extracellular DNA and histones indicated the release of neutrophil extracellular traps (NETs) [15]. Histones also significantly correlated with TF activity, providing further evidence for the presence of an immuno-thrombotic response (Figure 3).

We refrained from a direct quantification of LPS, since plasma components can interfere with the Limulus amoebocyte lysate assay. The elevated levels of LPS-binding protein (LBP) and sCD14, which both act as cofactors for the binding of LPS by toll-like receptors, however, provided strong evidence for the presence of LPS in the plasma samples. LBP significantly correlated with sCD14 and with the acute-phase protein CRP (Figure 3).

### 3.5. Markers of Endothelial Activation

To assess endothelial activation and alterations in vascular integrity, we quantified the vasoactive factors angiopoietin-1 (Ang-1) and Ang-2 as well as endothelial cell-specific molecule-1 (ESM-1, endocan), a proteoglycan mainly expressed by lung endothelial cells, whose secretion into the bloodstream is upregulated by proinflammatory cytokines and LPS [25]. ESM-1 levels were elevated as compared to reference values (Table 2) but did not correlate with any of the inflammatory parameters (Figure 1) [26, 27]. Both Ang-2 levels and the ratio of Ang-2/Ang-1 were substantially increased in our patient cohort, indicating increased vascular permeability. The Ang-2/Ang-1 ratio of 22 was very high as compared to previously published studies, which reported Ang-2/Ang-1 ratios of 5 for septic shock patients [28–30]. Moreover, we have found moderate positive correlations for Ang-2 and HMGB-1 (*r* = 0.551, *p* = 0.003). Extracellular DNA correlated with Ang-2 (*r* = 0.491, *p* = 0.007) and VEGF (*r* = 0.419, *p* = 0.024). VEGF also showed a positive correlation with HMGB-1 (*r* = 0.484, *p* = 0.011).

### 3.6. Depletion of Inflammatory Mediators by High Cut-Off *vs.* Standard Dialysis

The clinical characteristics of sepsis patients randomized to the treatment groups EMiC2 and AV1000S at baseline are shown in Table 4. Inflammatory mediator profiles in sepsis patients at baseline and after 48 h for EMiC2 and AV1000S are summarized in Table 5. Except for Gas6, Ang-2, TF, and TF activity, the baseline levels of inflammatory parameters were higher for patients randomized to the AV1000S group than to the EMiC2 group. This difference reached significance for IL-1*β*, IL-1ra, IL-6, IL-8, IL-15, IL-17, GM-CSF, PDGF, VEGF, and cystatin C (Table 5).

**Figure 4:**
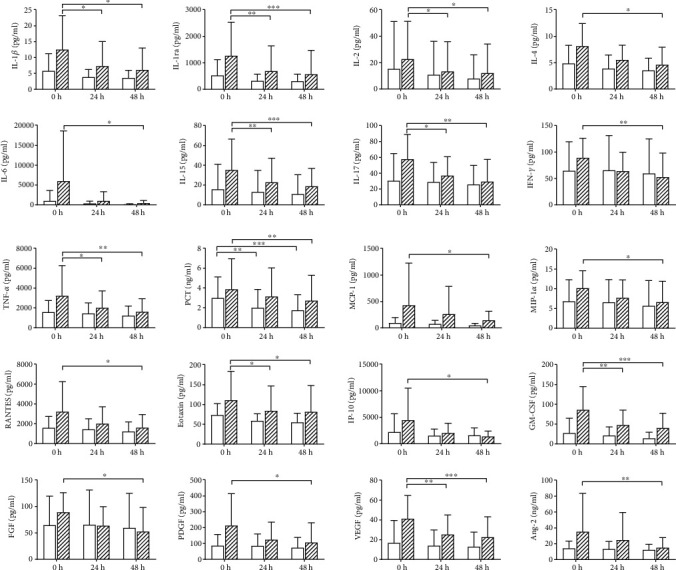
Effect of EMiC2 and AV1000S on inflammatory mediator concentrations over time (mean ± standard deviation, repeated measures two-way ANOVA using Bonferroni's multiple comparison test; *n* = 12 for EMiC2 (white bars), *n* = 10 for AV1000S (hatched bars); ^∗^*p* < 0.05, ^∗∗^*p* < 0.01, and ^∗∗∗^*p* < 0.001).

Treatment with AV1000S was associated with a significant decrease in IL-1*β*, IL-1ra, IL-2, IL-15, IL-17, TNF-*α*, eotaxin, GM-CSF, and VEGF over time (0 h *vs.* 24 h and 0 h *vs.* 48 h). Additionally, a significant reduction at 48 h was found for IL-4, IL-6, IFN-*γ*, PCT, MCP-1, MIP-1*α*, RANTES, IP-10, FGF, PDGF, and Ang-2. None of the inflammatory parameters decreased significantly over time for EMiC2, except for PCT (Figure 4). Despite the increased cut-off of the EMiC2 filter, albumin concentrations remained unaffected over time (data not shown).

## 4. Discussion

The characterization of inflammatory mediator profiles and their dynamic alterations can provide a basis for the timely prediction of the clinical course of sepsis and for the improved management of sepsis patients [5, 31, 32].

Here, we characterized a panel of 44 parameters in septic plasma samples, including cytokines, chemokines, growth factors, damage-associated molecular patterns, endothelial activation markers, and coagulation-related factors to depict the molecular mechanisms underlying sepsis on multiple levels. The study population included 30 patients suffering from acute renal failure in the course of sepsis, who received CVVHD with either high cut-off or standard filters. In line with previous studies, mediator concentrations in the study population at baseline (initiation of dialysis) ranged widely, despite thoroughly defined inclusion criteria [33–36]. The overall mortality was 56.7%, which was high compared to previously published studies [34, 36, 37]. Together with several other parameters, particularly the extremely high ratio of Ang-2/Ang-1 (see below), this indicated the severity of sepsis in our study population.

The strong correlations between IL-1*β*, IL-1ra, IL-4, IL-6, IL-8, IL-10, IL-15, IFN-*γ*, TNF-*α*, MCP-1, G-CSF, and GM-CSF provided evidence for the recruitment of innate immune cells (IL-8, IL-15, MCP-1, G-CSF, and GM-CSF). In line with previous studies [38, 39], they further indicated the presence of a proinflammatory immune response (IL-1*β*, IL-6, TNF-*α*, and IFN-*γ*), with a coexisting anti-inflammatory reaction (IL-1ra, IL-4, and IL-10). In particular, positive correlations between IL-6, IL-8, IL-10, MCP-1, MIP-1*β*, IFN-*γ*, and GM-CSF support previous findings [39].

We refrained from the quantification of LPS in plasma samples, since a number of plasma components as well as anticoagulants can interfere with the Limulus amoebocyte lysate assay. However, the elevated plasma concentrations of LBP and sCD14 pointed to the presence of LPS [40], as LBP and sCD14 are released to reduce or inhibit the response to LPS by limiting its interaction with Toll-like receptor- (TLR-) 4 [41, 42]. In addition to its association with sCD14, LBP also correlated with the acute-phase protein CRP. There is accumulating evidence that CRP, beyond its role as a marker for infection, can be actively involved in the propagation of inflammation, particularly when bound to extracellular vesicles released from activated platelets. Moreover, recent data indicate that patients with high CRP levels, together with elevated IL-6 and PCT, are more likely to experience severe complications due to the cytokine storm associated with coronavirus disease (COVID-19) [43].

We included extracellular DNA, histones, and HMGB-1 in our analysis, as they represent well-characterized DAMPs whose release is critical in sepsis [15]. All three factors were elevated and strongly correlated with each other, indicating neutrophil activation with NET formation [44–47], which can induce collateral damage to the host and potentiate tissue damage and thrombosis. NET-associated histones, in particular, display direct cytotoxic effects on eukaryotic cells, exert proinflammatory roles upon their release into the extracellular environment, and can contribute to endothelial barrier dysfunction [48–53]. They can trigger a procoagulant phenotype in endothelial cells and stimulate TF expression in a dose-dependent manner [54, 55], as also evidenced by the correlation of histone levels and TF activity in our study population. Our finding that nonsurvivors showed almost 30-fold higher baseline histone levels than survivors confirmed the significant contribution of histones to cellular injury and multiple organ failure and their potential predictive value for the clinical course of sepsis [56–58].

Next to immunothrombosis, microvascular endothelial dysfunction is a fundamental mechanism in sepsis. Considering this, our analysis included ESM-1, Ang-1, Ang-2, and VEGF as markers of altered endothelial function. ESM-1, a proteoglycan mainly expressed by lung and kidney endothelial cells, is released in response to proinflammatory cytokines [25, 59, 60]. It can block the interaction of monocytes with activated endothelial cells by impeding the binding of the monocytic integrin LFA-1 to endothelial ICAM-1 [61]. Accordingly, we have previously shown that septic plasma samples containing high ESM-1 levels failed to induce monocytic cell adhesion to endothelial cells *in vitro* [22].

Angiopoietins are essential for vasculogenesis and vascular stability and have been implicated in endothelial dysfunction in sepsis. Ang-1 promotes stabilization and maturation of new blood vessels, whereas Ang-2 can either promote VEGF-induced angiogenesis or destabilize blood vessels in a context-dependent fashion. Increased Ang-2/Ang-1 ratios have been described in sepsis and have been associated with disease severity and poor outcome [29, 62], as confirmed by the high mortality and the extraordinarily high Ang-2/Ang-1 ratio in our study population.

Patients enrolled in this study received CVVHD with high cut-off filters or with standard filters [21]. After randomization, baseline levels of most inflammatory mediators were higher for the standard filter group *vs.* the high cut-off filter group, and this difference was statistically significant for a number of factors. Clearly, these different baseline mediator levels are linked to the relatively small sample size of our study and limit conclusions with regard to differences in mediator depletion in the two filter groups. As a further limitation, the panel of parameters evaluated in this study cannot be immediately used for bedside monitoring, but our findings may contribute to the identification of smaller mediator panels to support early diagnosis of sepsis.

In conclusion, the analysis of a panel of inflammation-related parameters, including cytokines, chemokines, damage-associated molecular patterns, procoagulant factors, and markers of endothelial activation, highlights the high level of heterogeneity in sepsis. The finding that extracellular histones were significantly elevated in nonsurvivors as compared to survivors emphasizes the diagnostic and prognostic potential of circulating histones and nucleosomes. Sepsis patients may benefit from antihistone agents, such as nonanticoagulant heparin or hemoperfusion devices containing beads functionalized with heparin (Seraph 100 Microbind Affinity Blood Filter, ExThera Medical, Martinez, CA).

Finally, the findings of this study, specifically the wide concentration ranges of mediators in individual patients as well as the lack of consistency between clinical scores and biomarker levels, emphasize the current challenge of identifying distinct clinical phenotypes and of trial design and interpretation in sepsis patients [63].

## Figures and Tables

**Table 1:** Clinical characteristics of study patients at baseline.

Characteristics	All patients (*n* = 30)
Age (years)	62.3 ± 18.3 (26-89)
Gender, male	19 (63%)
SAPS III	68.4 ± 12.5 (45-107)
TISS	39.5 ± 7.1 (26-57)
Leukocyte count (×10^3^/*μ*l)	15.0 ± 8.6 (2-35)
Comorbidities, number (% of total)	
Cardiovascular	18 (60%)
Pulmonary	7 (23%)
Hepatitis/pancreatitis	1 (3%)
Neurological	9 (30%)
Renal	5 (17%)
Diabetes	9 (30%)
Primary site of infection, number (% of total)	
Lung	1 (3%)
Abdomen	10 (33%)
Blood	7 (23%)
Urinary tract	3 (10%)
Other	7 (23%)
Unknown	2 (7%)

Data are represented as mean ± standard deviation (range) or *n* (%). SAPS III: Simplified Acute Physiology Score III; TISS: Therapeutic Intervention Scoring System.

**Table 2:** Biomarker profiles in sepsis patients at baseline (*n* = 30).

Parameter		Median	IQR (25^th^-75^th^ quartile)
Inflammatory mediators			
IL-1*β*	(pg/ml)	6.82	4.37-14.55
IL-1ra	(pg/ml)	670.66	266.44-1,231.08
IL-2	(pg/ml)	7.62	1.12-32.42
IL-4	(pg/ml)	6.36	4.82-10.11
IL-5	(pg/ml)	19.94	15.94-35.56
IL-6	(pg/ml)	277.02	103.65-1,091.49
IL-7	(pg/ml)	8.14	4.64-22.54
IL-8	(pg/ml)	104.56	53.26-198.48
IL-9	(pg/ml)	15.76	8.62-34.04
IL-10	(pg/ml)	28.48	10.59-65.04
IL-12	(pg/ml)	19.54	8.71-45.81
IL-13	(pg/ml)	6.68	0.84-11.68
IL-15	(pg/ml)	14.30	1.36-57.00
IL-17	(pg/ml)	49.22	19.65-86.74
IFN-*γ*	(pg/ml)	173.40	112.79-295.45
TNF-*α*	(pg/ml)	84.56	44.98-152.19
CRP	(mg/l)	208.15	164.26-283.67
PCT	(ng/ml)	3.00	0.78-4.36
LBP	(*μ*g/ml)	114.93	77.47-172.14
sCD14	(ng/ml)	3,159.00	2,728.00-3,721.00
sST2	(ng/ml)	372.36	173.64-589.74
Gas6	(ng/ml)	43.15	34.03-55.44
Chemokines			
MCP-1	(pg/ml)	125.72	49.97-385.93
MIP-1*α*	(pg/ml)	8.48	5.72-12.92
MIP-1*β*	(pg/ml)	120.94	96.09-200.34
RANTES	(pg/ml)	2,014.36	1,088.59-3,285.85
Eotaxin	(pg/ml)	95.78	59.67-116.16
IP-10	(pg/ml)	1,375.94	822.83-2,734.51
Growth factors			
G-CSF	(pg/ml)	161.60	70.82-637.97
GM-CSF	(pg/ml)	54.32	13.46-109.47
FGF	(pg/ml)	79.94	56.00-116.24
PDGF	(pg/ml)	118.70	68.22-218.79
VEGF	(pg/ml)	25.98	10.39-60.54
Endothelial activation markers			
ESM-1	(ng/ml)	6.00	3.86-12.95
Ang-1	(ng/ml)	0.59	0.43-1.22
Ang-2	(ng/ml)	13.85	7.85-33.21
Ang-2/Ang-1		22.99	8.96-61.99
Damage-associated molecular patterns			
ecDNA^a^	(ng/ml)	545.02	276.11-1,194.48
Histones^a^	(mg/ml)	6.60	0.45-24.67
HMGB-1^b^	(ng/ml)	3.70	2.6-6.91
Coagulation-related parameters			
TF	(pg/ml)	66.98	50.64-91.59
TF activity	(pM)	38.13	24.57-55.79
Others			
Cystatin C	(*μ*g/ml)	3.75	2.92-4.05
Albumin	(g/dl)	3.38	2.83-3.81

Data are represented as median and interquartile range (IQR, 25^th^-75^th^ quartile). ^a^*n* = 29; ^b^*n* = 27.

**Table 3:** Correlation analysis of inflammatory cytokines, chemokines, and growth factors (*n* = 30).

	IL-1*β*	IL-1ra	IL-4	IL-6	IL-8	IL-10	IL-15	IFN-*γ*	TNF-*α*	MCP-1	G-CSF	GM-CSF
IL-1*β*		0.787	0.835	0.788	0.581	0.692	0.771	0.963	0.936	0.719	0.687	0.775
IL-1ra			0.660	0.757	0.689	0.844	0.820	0.818	0.825	0.759	0.641	0.714
IL-4				0.723	0.630	0.696	0.721	0.852	0.824	0.652	0.682	0.802
IL-6					0.770	0.626	0.686	0.755	0.686	0.806	0.747	0.753
IL-8						0.591	0.607	0.581	0.539	0.782	0.762	0.666
IL-10							0.912	0.771	0.756	0.750	0.575	0.805
IL-15								0.817	0.807	0.780	0.650	0.849
IFN-*γ*									0.934	0.687	0.699	0.813
TNF-*α*										0.677	0.652	0.783
MCP-1											0.715	0.784
G-CSF												0.728
GM-CSF												

**Table 4:** Clinical characteristics of patients randomized to EMiC2 and AV1000S at baseline.

Characteristics	EMiC2 (*n* = 14)	AV1000S (*n* = 16)
Age (years)	61.7 ± 16.5 (26-81)	62.8 ± 20.3 (26-89)
Gender, male	6 (43%)	13 (81%)^∗^
SAPS III	67.7 ± 10.3 (48-85)	69.0 ± 14.4 (45-107)
TISS	40.6 ± 7.8 (30-57)	38.6 ± 6.4 (26-48)
Leukocyte count (×10^3^/*μ*l)	16.2 ± 7.8 (2-34)	14.0 ± 9.4 (2-35)
Nonsurvivors	8 (57.1%)	9 (56.3%)
Comorbidities, number (% of total)		
Cardiovascular	8 (57%)	10 (63%)
Pulmonary	1 (7%)	6 (38%)
Hepatitis/pancreatitis	1 (7%)	0 (0%)
Neurological	6 (43%)	3 (19%)
Renal	1 (7%)	4 (25%)
Diabetes	3 (21%)	6 (38%)
Primary site of infection, number (% of total)		
Lung	1 (7%)	0 (0%)
Abdomen	5 (36%)	5 (31%)
Blood	3 (21%)	4 (25%)
Urinary tract	1 (7%)	2 (13%)
Other	3 (21%)	4 (25%)
Unknown	1 (7%)	1 (6%)

Data are represented as mean ± standard deviation (range) or *n* (%). SAPS III: Simplified Acute Physiology Score III; TISS: Therapeutic Intervention Scoring System. ^∗^*p* = 0.030.

**Table 5:** Biomarker profiles in sepsis patients at baseline and after 48 h for EMiC2 and AV1000S.

Parameter		0 h		48 h	
		EMiC2 (*n* = 14)	AV1000S (*n* = 16)	EMiC2 (*n* = 12)	AV1000S (*n* = 10)
Inflammatory mediators					
IL-1*β*	(pg/ml)	5.92 (1.66-9.21)^∗^	8.80 (5.60-21.21)	4.12 (81.15-5.64)	4.12 (2.05-8.43)
IL-1ra	(pg/ml)	301.44 (112.64-1,082.40)^∗^	962.12 (422.33-2,706.33)	259.44 (168.56-383.78)	294.44 (142.33-533.06)
IL-2	(pg/ml)	1.12 (1.12-14.36)	16.88 (1.12-34.91)	1.12 (1.12-1.87)	1.12 (1.12-14.94)
IL-4	(pg/ml)	5.56 (2.53-8.25)	7.58 (5.21-10.62)	4.32 (0.81-5.56)	4.46 (1.72-6.87)
IL-5	(pg/ml)	17.48 (13.25-29.07)	23.22 (15.11-36.60)	16.40 (5.32-25.88)	15.30 (3.02-34.37)
IL-6	(pg/ml)	108.76 (43.38-693.86)^∗^	372.14 (256.94-8,914.32)	63.94 (25.22-279.45)	148.04 (36.39-476.18)
IL-7	(pg/ml)	7.12 (3.49-22.74)	10.14 (5.47-25.29)	10.14 (2.56-17.51)	7.04 (2.38-26.49)
IL-8	(pg/ml)	70.06 (35.18-128.29)^∗^	181.50 (71.64-309.90)	47.10 (35.52-60.96)	54.66 (45.07-79.99)
IL-9	(pg/ml)	10.58 (5.11-21.22)	24.50 (9.28-40.76)	10.76 (3.67-14.46)	21.60 (4.84-50.33)
IL-10	(pg/ml)	10.76 (3.12-60.99)	44.94 (15.64-90.29)	11.76 (2.70-19.45)	22.58 (2.15-40.24)
IL-12	(pg/ml)	12.90 (5.59-46.92)	24.70 (17.40-45.64)	14.40 (3.67-34.50)	16.82 (4.85-53.85)
IL-13	(pg/ml)	4.58 (0.49-10.39)	7.48 (2.32-13.69)	2.90 (0.49-11.07)	5.34 (0.49-11.41)
IL-15	(pg/ml)	2.18 (1.36-42.09)^∗^	30.52 (11.05-69.96)	2.20 (1.36-6.54)	14.92 (1.36-36.92)
IL-17	(pg/ml)	21.98 (1.76-59.59)^∗^	61.36 (33.91-95.71)	20.96 (2.29-44.40)	16.22 (5.30-56.03)
IFN-*γ*	(pg/ml)	141.98 (52.02-236.75)	235.00 (132.64-367.44)	106.74 (11.40-175.60)	111.46 (31.02-246.25)
TNF-*α*	(pg/ml)	67.24 (21.35-127.49)	130.36 (66.29-258.62)	40.10 (10.36-69.18)	47.90 (28.27-121.97)
CRP	(mg/l)	182.75 (152.39-309.13)	225.20 (170.16-279.81)	194.59 (122.48-259.60)	211.55 (143.30-266.25)
PCT	(ng/ml)	2.64 (0.63-4.19)	3.14 (1.22-5.84)	1.26 (0.50-2.63)	1.86 (0.50-4.55)
LBP	(*μ*g/ml)	111.70 (71.10-178.66)	117.53 (77.75-175.31)	94.64 (67.16-178.19)	98.13 (59.89-109.24)
sCD14	(ng/ml)	3,141.50 (2,652.75-3,596.50)	3,256.50 (2,741.00-4,153.75)	2,835.50 (2,460.75-3,211.00)	2,812.00 (2,103.25-3,926.25)
sST2	(ng/ml)	311.56 (137.45-501.29)	460.20 (291.00-616.22)		
Gas6	(ng/ml)	49.82 (30.53-56.13)	41.37 (37.88-51.81)	39.73 (34.45-53.00)	37.83 (30.14-55.57)
Chemokines					
MCP-1	(pg/ml)	77.84 (18.58-196.61)	217.92 (58.49-452.11)	30.30 (20.34-65.31)	98.76 (43.46-169.94)
MIP-1*α*	(pg/ml)	7.40 (3.60-13.36)	9.76 (7.45-12.92)	5.72 (0.06-8.84)	6.44 (1.34-10.68)
MIP-1*β*	(pg/ml)	113.96 (86.25-136.21)	140.10 (99.45-250.56)	87.68 (66.20-118.70)	87.80 (69.61-206.18)
RANTES	(pg/ml)	1,761.46 (358.78-2,779.92)	2,034.30 (1,120.56-4,104.55)	928.68 (436.46-2,185.56)	1,158.44 (408.50-3,027.76)
Eotaxin	(pg/ml)	70.56 (49.43-105.12)	101.08 (76.99-190.60)	50.36 (45.00-69.06)	68.12 (36.38-97.11)
IP-10	(pg/ml)	1,256.80 (863.08-2,267.60)	1,572.08 (698.69-3,027.45)	1,230.46 (602.92-2,062.71)	853.12 (530.21-2,442.66)
Growth factors					
G-CSF	(pg/ml)	96.38 (37.36-441.77)	185.96 (150.13-645.11)	94.88 (72.43-136.79)	109.36 (75.29-137.63)
GM-CSF	(pg/ml)	16.54 (0.78-95.23)^∗^	83.54 (31.07-159.81)	8.32 (0.78-27.19)	28.84 (4.77-74.65)
FGF	(pg/ml)	61.88 (21.93-107.02)	93.12 (66.33-119.76)	49.48 (5.12-82.51)	51.00 (4.09-95.82)
PDGF	(pg/ml)	91.70 (45.13-187.68)^∗^	152.26 (83.73-266.76)	59.62 (29.91-98.59)	63.66 (15.67-146.77)
VEGF	(pg/ml)	9.84 (1.80-51.50)^∗^	44.02 (18.84-61.46)	7.48 (1.80-22.88)	20.36 (4.92-36.96)
Endothelial activation markers					
ESM-1	(ng/ml)	4.76 (3.75-7.90)	7.24 (4.11-21.60)	5.86 (2.81-12.89)	16.39 (4.69-44.79)
Ang-1	(ng/ml)	0.56 (0.29-1.02)	0.73 (0.50-1.31)	0.36 (0.14-0.77)	0.62 (0.14-1.26)
Ang-2	(ng/ml)	15.21 (6.42-27.03)	13.03 (8.93-56.98)	10.42 (8.06-18.55)	10.09 (7.96-18.37)
Ang-2/Ang-1		27.98 (10.92-45.96)	19.10 (8.47-128.80)	37.85 (9.75-133.65)	37.87 (7.01-110.56)
Damage-associated molecular patterns					
ecDNA	(ng/ml)	441.78 (244.90-690.28)^a^	657.27 (397.96-1,542.62)		
Histones	(mg/ml)	0.60 (0.46-22.29)^a^	7.47 (0.40-79.90)		
HMGB-1	(ng/ml)	2.99 (2.55-6.63)^a^	4.53 (2.89-12.53)^b^	2.99 (2.50-4.56)^b,^^∗^	5.13 (3.26-9.16)^c^
Coagulation-related parameters					
TF	(pg/ml)	71.12 (42.24-108.66)	65.90 (50.96-79.93)		
TF activity	(pM)	46.73 (23.69-66.11)	33.10 (24.63-45.34)		
Others					
Cystatin C	(*μ*g/ml)	3.26 (2.60-4.00)^∗^	3.91 (3.61-4.20)	2.68 (2.52-3.21)^∗^	3.46 (3.03-3.86)
Albumin	(g/dl)	3.42 (2.50-3.86)	3.35 (2.89-3.77)	3.55 (3.11-3.88)	3.13 (2.69-3.34)

Data are represented as median (25^th^-75^th^ quartile). ^a^*n* = 13; ^b^*n* = 14; ^c^*n* = 8. Differences in inflammatory mediator concentrations between filter groups at baseline and after 48 h were analyzed by the nonparametric Mann-Whitney test (^∗^*p* < 0.05).

## Data Availability

All authors confirm that all relevant data are included in the article. Additional statistical data are available from the corresponding author upon request. Data supporting the findings of this study are provided as supplementary information accompanying this paper.

## Supplementary Materials

Supplementary Materials Supplementary Table 1: Clinical characteristics of survivors and nonsurvivors at baseline. Supplementary Table 2: Biomarker profiles in survivors and nonsurvivors at baseline.
