# Supplementary material for: Analysis of Inflammatory Mediator Profiles in Sepsis Patients Reveals That Extracellular Histones Are Strongly Elevated in Nonsurvivors
Source: Mediators Inflamm. 2021 Mar 17;2021:8395048. doi: 10.1155/2021/8395048 (PMC7994100; doi:10.1155/2021/8395048)
Supplement: Supplementary Materials — Supplementary Table 1: Clinical characteristics of survivors and nonsurvivors at baseline. Supplementary Table 2: Biomarker profiles in survivors and nonsurvivors at baseline. [file 8395048.f1.doc]

**Supplementary Material**

**Supplementary Table 1:** Clinical characteristics of survivors and nonsurvivors at baseline.

| **Characteristics** | **All patients (n=30)** | | **Survivors (n=13)** | | **Nonsurvivors (n=17)** | |
| --- | --- | --- | --- | --- | --- | --- |
| Age (years)  Gender, male  SAPS III  TISS  Leukocyte count (x 10^3^/µl) | 62.3±18.3 (26-89)  19 (63%)  68.4±12.5 (45-107)  39.5±7.1 (26-57)  15.0±8.6 (2-35) | | 60.5±19.6 (26-89)  7 (54%)  64.8±13.0 (45-85)  41.2±7.7 (30-57)  16.7±7.6 (4-34) | | 63.7±17.7 (26-83)  12 (71%)  71.2±11.7 (56-107)  38.3±6.5 (26-47)  13.7±9.3 (2-35) | |
| ***Co-morbidities, number (% of total)*** | |  | |  | |  |
| Cardiovascular  Pulmonary  Hepatitis/pancreatitis  Neurological  Renal  Diabetes | 18 (60%)  7 (23%)  1 (3%)  9 (30%)  5 (17%)  9 (30%) | | 8 (62%)  2 (15%)  1 (8%)  3 (23%)  3 (23%)  4 (31%) | | 10 (59%)  5 (29%)  0 (0%)  6 (35%)  2 (12%)  5 (29%) | |
| ***Primary site of infection, number (% of total)*** | | |  | |  | |
| Lung  Abdomen  Blood  Urinary Tract  Other  Unknown | 1 (3%)  10 (33%)  7 (23%)  3 (10%)  7 (23%)  2 (7%) | | 0 (0%)  4 (31%)  3 (23%)  1 (8%)  5 (39%)  0 (0%) | | 1 (6%)  6 (35%)  4 (24%)  2 (12%)  2 (12%)  2 (12%) | |

Data are represented as mean±standard deviation (range) or n (%). SAPS III: Simplified Acute Physiology Score III; TISS: Therapeutic Intervention Scoring System.

**Supplementary Table 2: Biomarker profiles in survivors and nonsurvivors at baseline.**

| **Parameter** | **All patients (n=30)** | **Survivors (n=13)** | **Nonsurvivors (n=17)** |
| --- | --- | --- | --- |
| ***Inflammatory mediators*** | |  |  |
| IL-1β [pg/ml] | 6.82 (4.37-14.55) | 6.20 (3.90-15.90) | 7.84 (4.84-15.70) |
| IL-1ra [pg/ml] | 670.66 (266.44-1,231.08) | 540.00 (266.44-1,044.08) | 907.76 (238.46-1,627.56) |
| IL-2 [pg/ml] | 7.62 (1.12-32.42) | 1.12 (1.12-31.70) | 8.56 (1.12-32.84) |
| IL-4 [pg/ml] | 6.36 (4.82-10.11) | 5.56 (4.62-8.20) | 7.40 (5.36-11.22) |
| IL-5 [pg/ml] | 19.94 (15.94-35.56) | 22.32 (16.98-33.40) | 17.48 (14.32-38.60) |
| IL-6 [pg/ml] | 277.02 (103.65-1,091.49) | 250.48 (128.72-911.28) | 277.72 (99.22-4,532.86) |
| IL-7 [pg/ml] | 8.14 (4.64-22.54) | 7.12 (3.82-15.76) | 13.04 (4.36-26.92) |
| IL-8 [pg/ml] | 104.56 (53.26-198.48) | 89.00 (55.34-172.96) | 142.72 (38.92-305.04) |
| IL-9 [pg/ml] | 15.76 (8.62-34.04) | 13.16 (8.52-35.46) | 19.76 (8.70-36.92) |
| IL-10 [pg/ml] | 28.48 (10.59-65.04) | 16.60 (11.10-46.14) | 51.00 (8.74-87.88) |
| IL-12 [pg/ml] | 19.54 (8.71-45.81) | 28.36 (9.92-46.92) | 19.24 (8.42-40.38) |
| IL-13 [pg/ml] | 6.68 (0.84-11.68) | 10.16 (1.16-14.64) | 6.36 (0.49-8.76) |
| IL-15 [pg/ml] | 14.30 (1.36-57.00) | 12.56 (4.00-47.90) | 30.36 (1.36-59.16) |
| IL-17 [pg/ml] | 49.22 (19.65-86.74) | 49.72 (22.52-79.46) | 48.72 (18.84-99.90) |
| IFN-γ [pg/ml] | 173.40(112.79-295.45) | 160.12 (104.24-259.08) | 220.48 (116.32-313.04) |
| TNF-α [pg/ml] | 84.56 (44.98-152.19) | 76.88 (44.00-164.48) | 88.40 (49.82-152.20) |
| CRP [mg/l] | 208.15 (164.26-283.67) | 217.90 (171.90-282.10) | 178.75 (155.55-286.97) |
| PCT [ng/ml] | 3.00 (0.78-4.36) | 3.15 (1.62-5.69) | 2.87 (0.59-3.97) |
| LBP [µg/ml] | 114.93 (77.47-172.14) | 106.50 (75.01-172.10) | 123.00 (77.32-183.03) |
| sCD14 [ng/ml] | 3,159.00 (2,728.00-3,721.00) | 3,456.00 (3,064.50-3,754.00) | 2,780.00 (2,571.50-4,051.50) |
| sST2 [ng/ml] | 372.36 (173.64-589.74) | 380.99 (188.62-518.33) | 341.27 (170.53-614.76) |
| Gas6 [ng/ml] | 43.15 (34.03-55.44) | 42.75 (34.93-54.97) | 44.93 (33.29-59.79) |
| ***Chemokines*** | |  |  |
| MCP-1 [pg/ml] | 125.72 (49.97-385.93) | 63.64 (42.18-294.68) | 158.24 (42.66-466.42) |
| MIP-1α [pg/ml] | 8.48 (5.72-12.92) | 8.32 (5.88-12.36) | 9.68 (5.48-14.10) |
| MIP-1β [pg/ml] | 120.94 (96.09-200.34) | 118.20 (105.38-146.12) | 132.36 (80.96-240.30) |
| RANTES [pg/ml] | 2,014.36 (1,088.59-3,285.85) | 2068.60 (1,198.80-2,618.34) | 1,515.64 (1,087.18-3,785.36) |
| Eotaxin [pg/ml] | 95.78 (59.67-116.16) | 93.88 (57.38-102.56) | 102.16 (57.70-187.52) |
| IP-10 [pg/ml] | 1,375.94 (822.83-2,734.51) | 1,014.20 (744.68-1,669.66) | 1,709.92 (895.72-3,179.64) |
| ***Growth factors*** |  |  |  |
| G-CSF [pg/ml] | 161.60 (70.82-637.97) | 167.76 (75.74-851.12) | 155.44 (58.96-462.28) |
| GM-CSF [pg/ml] | 54.32 (13.46-109.47) | 39.68 (18.06-95.92) | 77.68 (0.78-162.20) |
| FGF [pg/ml] | 79.94 (56.00-116.24) | 92.52 (63.28-130.62) | 67.76 (54.44-111.12) |
| PDGF [pg/ml] | 118.70 (68.22-218.79) | 119.76 (67.14-194.94) | 117.64 (67.68-262.12) |
| VEGF [pg/ml] | 25.98 (10.39-60.54) | 20.64 (13.86-53.32) | 39.84 (3.24-62.86) |
| ***Endothelial activation markers*** | |  |  |
| ESM-1 [ng/ml] | 6.00 (3.86-12.95) | 6.41 (3.65-9.82) | 5.59 (4.16-16.95) |
| Ang-1 [ng/ml] | 0.59 (0.43-1.22) | 0.58 (0.44-0.89) | 0.67 (0.43-1.57) |
| Ang-2 [ng/ml] | 13.85 (7.85-33.21) | 13.40 (7.83-25.93) | 14.31 (7.63-58.81) |
| Ang-2/Ang-1 | 22.99 (8.96-61.99) | 20.03 (8.30-58.74) | 25.95 (10.04-118.48) |
| ***Damage-associated molecular patterns*** | |  |  |
| ecDNA [ng/ml] | 545.02 (276.11-1,194.48)^a^ | 460.98 (219.69-750.30) | 594.24 (342.74-1,943.28)^d^ |
| Histones [mg/ml] | 6.60 (0.45-24.67)^a^ | 0.55 (0.26-10.69) | 15.38 (2.83-89.21)^d, *^ |
| HMGB-1 [ng/ml] | 3.70 (2.60-6.91)^b^ | 2.99 (2.50-3.77)^c^ | 5.70 (2.74-13.14)^d^ |
| ***Coagulation-related parameters*** | |  |  |
| TF [pg/ml] | 66.98 (50.64-91.59) | 65.44 (46.66-90.06) | 71.90 (55.20-96.92) |
| TF activity [pM] | 38.13 (24.57-55.79) | 26.68 (22.59-49.34) | 41.95 (27.44-60.68) |
| ***Others*** |  |  |  |
| Cystatin C [µg/ml] | 3.75 (2.92-4.05) | 3.88 (2.91-4.01) | 3.70 (2.94-4.08) |
| Albumin [g/dl] | 3.38 (2.83-3.81) | 3.50 (2.94-3.87) | 3.34 (2.77-3.82) |

Data are represented as median and interquartile range (IQR, 25^th^- 75^th^ quartile). ^a^ n=29, ^b^ n=27, ^c^ n=11, ^d^ n=16. Differences in inflammatory mediator concentrations between filter groups at baseline between survivors and nonsurvivors were analyzed by the non-parametric Mann-Whitney test (* *p*=0.025).
